# In Vitro Gastrointestinal Digestion and Gut Fermentation of Atlantic Forest Fruit‐Flavored Kombucha: Gut Microbiota Dynamics and Bioactive Compound Bioaccessibility

**DOI:** 10.1002/mnfr.70620

**Published:** 2026-09-30

**Authors:** Kawany de Faria Forato, Vinícius dos Santos Domingues, Eduardo Lolato, Silvia Turroni, Semeh Bejaoui, Federica D'Amico, Volker Behrends, Solidea Amadei, Asia Pizzi, Giselle Nobre Costa, Adele Costabile, Karla Bigetti Guergoletto

**Affiliations:** ^1^ Department of Food Science and Technology State University of Londrina Londrina Brazil; ^2^ Unit of Microbiome Science and Biotechnology, Department of Pharmacy and Biotechnology University of Bologna Bologna Italy; ^3^ IRCCS Azienda Ospedaliero‐Universitaria di Bologna Bologna Italy; ^4^ Centre For Innovation in Genomics and Microbiome Sciences, School of Medicine and Biosciences University of West London London UK; ^5^ Department of Agricultural and Food Sciences, Campus of Food Science, Alma Mater Studiorum University of Bologna Cesena Italy; ^6^ Beneficial Microbes Laboratory, Department of Pharmacy and Biotechnology University of Bologna Bologna Italy; ^7^ School of Life and Health Sciences University of Roehampton London UK

**Keywords:** bioaccessibility, gut fermentation, kombucha

## Abstract

Fermented foods are sources of highly bioavailable nutrients and compounds with health benefits. This exploratory study evaluated compound bioaccessibility, microbial survival and the effects of kombucha with Atlantic Forest fruits (jaboticaba, grumixama, juçara) on gut microbiota in healthy individuals. Samples underwent simulated digestion and 24 h fermentation with human fecal microbiota. Microbiota composition was analyzed by 16S rRNA amplicon sequencing, and SCFAs and BCFA were quantified. Yeasts, acetic acid bacteria, and lactic acid bacteria remained viable after digestion. Flavored kombucha (FK) showed high antioxidant activity. After 24 h, SCFAs increased, particularly acetate in FK; natural kombucha showed moderate effects, while tea and commercial kombucha caused minimal changes. Time was a major driver of variation in alpha and beta diversity, with the former declining especially with FK. The main taxonomic shifts included an increase in *Enterobacteriaceae* and a reduction in some beneficial taxa, with formulation‐dependent specific modulation.

## Graphical abstract

Atlantic Forest fruit‐flavored kombucha affects gut microbiota and bioactive compounds during simulated digestion and gut fermentation. Beneficial microorganisms survived digestion, while flavored kombucha showed high antioxidant activity. Fermentation increased short‐chain fatty acids, especially acetate. Gut microbiota changed over time, with effects depending on kombucha formulation.

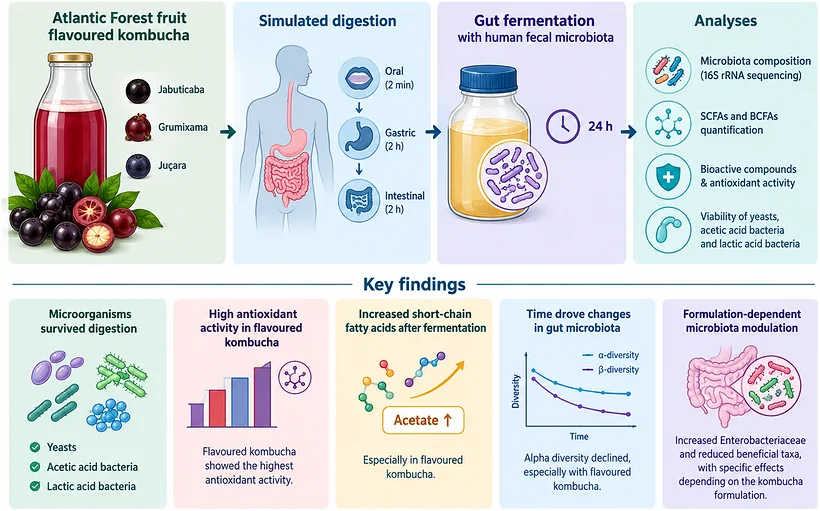

**Abbreviations**
AAB: acetic acid bacteria
BCFA: branched‐chain fatty acids
FK: Flavored kombucha
LAB: lactic acid bacteria
SCOBY: symbiotic culture of bacteria and yeast

## 1 Introduction

The gut microbiota plays a key role in human health, influencing metabolism, the immune system and the nervous system [1]. This is made possible by the plethora of bioactive compounds produced by gut microbes, also through diet metabolism. These compounds enter the bloodstream and reach different organs in the body [2]. Modulating the microbiome is becoming an important adjunct to many preventive and therapeutic strategies in several clinical settings. This involves using prebiotics, probiotics, postbiotics, faecal microbiota transplants, or ad hoc synthetic consortia [3, 4].

Fermented foods are recognized as sources of probiotic microorganisms that provide benefits beyond basic nutrition [5]. Fermentation transforms food components, enhancing nutritional value and generating bioactive compounds. In the gut, the resident microbiota further metabolizes them into beneficial substances. Their combined interaction with the gut microbiota and immune system promotes positive physiological effects. Thus, fermented foods are proposed as dietary strategies to help counter reduced microbiome diversity and inflammation linked to dysbiosis [6].

Kombucha is a fermented beverage, produced by fermenting sweetened black or green tea (*Camellia sinensis*) with a symbiotic culture of acetic and lactic acid bacteria and yeast (SCOBY). It contains polyphenols, catechins, flavonoids, amino acids, vitamins, enzymes, and organic acids, giving it a fizzy, slightly sweet, acidic profile. Its low pH inhibits harmful microorganisms, while polyphenols enhance antioxidant activity. Recent studies suggest potential anti‐hyperglycaemic, anti‐inflammatory, and anti‐obesity effects [7, 8, 9, 10]. The enrichment of a beverage as Kombucha with native fruits from the Brazilian Atlantic Forest may offer additional functional benefits due to the unique and rich phytochemical composition of these fruits. Therefore, investigating the bioaccessibility of these compounds and the impact in gastrointestinal environment is essential to better understand their potential health effects and to support the development of novel functional beverages based on underexplored biodiversity.

In the present exploratory study, we investigated the bioaccessibility of bioactive compounds, survival rate of microorganisms, and effects of kombucha flavored with native fruits from the Brazilian Atlantic Forest, jaboticaba (*Myrciaria cauliflora*), grumixama (*Eugenia brasiliense*), and juçara (*Euterpe edulis*), on the gut microbiota composition and metabolic profile of healthy individuals using a gut model system.

## 2 Experimental Section

The study was approved by the University of Roehampton Research Ethics Committee (LSC 24/422) and conducted in accordance with the Declaration of Helsinki. The sample size for in vitro gastrointestinal digestion was based on previous studies in this area [11].

### 2.1 Materials and Methods

The native fruits from Atlantic Forest were purchased from different places in Brazil; jaboticaba (*M. cauliflora)* from Bicudo Alimentos Congelados (São Paulo, Brazil 23°32'15.3“S 46°44'04.5”W), grumixama (*E. brasiliense*) from Agroecology Site English Camp—Immersion and Camping (Itapetininga, São Paulo, Brazil 23°29'44.8“S 47°56'56.4”W) and juçara (*E. edulis*) from BIMINI Farm (Rolândia, Paraná, Brazil 23°18′38″S 51°22′10″W). The green tea (*C. sinensis*) was produced by YAMAMOTOYAMA (Brazil). The SCOBY for kombucha fermentation was purchased on the website *ciadosfermentados.com.br/collections/scoby‐de‐kombucha*.

Fruit pulps were freeze‐dried, packed in polyethylene bags, and stored at a refrigerated temperature (4°C ± 1°C). The SCOBY was kept and transported in a vacuum‐packed container, then kept at 4°C until use. Green tea and native fruit flavoring were selected to enhance kombucha's antioxidant capacity through tea polyphenols and fruit bioactive compounds [12, 13].

#### 2.1.1 Kombucha Inoculum, Fruit‐Flavoring and Fermentation Process

Kombucha was produced according to Jayabalan et al. [14], involving the steps: first fermentation, flavouring, second fermentation, and bottling. For first fermentation, 1 L of green tea infusion with 5% (w/v) sugar was prepared, cooled to 20°C, and added with 10% (v/v) of the starter (previously fermented kombucha), and 2.5% (w/v) of SCOBY. Tea was incubated at 25°C for 7 days. After fermentation, filtration was performed to remove solid residues. Freeze‐dried pulp was reconstituted and used to flavor the fermented tea, by adding 10% (v/v) pulp (a mixture of jaboticaba, grumixama, and juçara pulp in a 1:1:1 ratio). The container was tightly closed and refrigerated at 10°C for 24 h. Then, kombucha was bottled to allow natural carbonation. The second fermentation occurred naturally in the bottle for a further 7 days at 4°C. After this period, the beverage “Flavored Kombucha” (FK) was ready and kept refrigerated until use. The same procedure was followed to produce “Natural Kombucha”, except for the fruit pulp.

A commercially available carbonated kombucha mixed berry flavored was also used in the analysis in the gut model system, as a reference of a commercial sample, together with the positive and negative control. The beverage was purchased from a local market in London, UK.

### 2.2 In Vitro Gastrointestinal Digestion

Three batches FK was submitted to in vitro digestion following described method by Minekus et al. [15], composed by oral, gastric, and intestinal phases. The sample size was n = 3, representing independent biological replicates. Faecal samples (∼20 g) were provided by three healthy volunteers who had not taken any medications or supplements prior to the study. All participants gave written informed consent, confirming that their samples would be used exclusively for experiments conducted in dynamic batch culture glass systems designed to simulate colonic conditions. Doses of the sample used followed the protocol and do not represent toxicity when compared to physiological conditions. At the end of each digestion phase, samples were collected for subsequent microbiological analysis. Additionally, an aliquot of each sample was centrifuged at 12 000 rpm for 10 min, and the supernatant was stored at ‐80°C for later bioaccessibility analysis of bioactive compounds.

### 2.3 Microbiological Analysis of Kombucha

Total viable counts of bacteria and yeasts in kombucha were determined as described by Sievers et al. [16] and Cvetković et al. [17]. For yeast enumeration, acidified Potato Dextrose Agar (PDA‐AC, Kasvi) was used, supplemented with 1 mL/L of 10% (v/v) tartaric acid. Plates were incubated at 25°C for 72 h. Total viable count of acetic AAB was determined using MYP agar medium, containing 25 g/L mannitol, 5 g/L yeast extract, and 3 g/L peptone, applied with a double‐layer technique. This consisted of an initial layer of 0.5% agar, followed by a second layer containing 1.0% agar. Plates were incubated at 30°C for 72 h, according to Entani et al. [18, 19]. LAB viable counts were obtained using the pour plate technique with de Man, Rogosa, and Sharp (MRS, Kasvi) agar, with incubation at 37°C for 48 h. Plating was performed in triplicate from serial dilutions of the FK, as well from samples obtained from the in vitro digestion phases.

### 2.4 Bioaccessibility of Bioactive Compounds

The bioaccessible fraction was estimated based on the amount of compounds released from the sample during in vitro digestion that remained soluble in the medium representing the human gastrointestinal tract. Following equation was applied:

$$Bioaccessibility\;\left(\%\right)=\frac{BC\;intestinal}{BC\;beverage}x100\;\left(\mathrm{Macedo}\;\mathrm{et}\;\mathrm{al}.\;\left[19\right]\right)$$

where “BC intestinal” represents the concentration of compound quantified in the sample after digestion, and “BC beverage” represents the concentration of compound in the beverage before digestion. Bioaccessible fractions were determined for antioxidant capacity and phenolic profile, as described in the following subsections.

#### 2.4.1 Anthocyanin

The anthocyanin content was quantified according to the method described by Barbosa et al. [20]. Absorbance was measured using a spectrophotometer at two wavelengths: 520 nm and 700 nm. Values were applied to the following formula:

A=(Aλvis−max−A700)pH1.0−(Aλvis−max−A700)pH4.5

where: A = absorbance of the diluted sample; A *𝜆*vis‐max = absorbance measured at 520 nm; A700 = absorbance measured at 700 nm.

Anthocyanin content was calculated as follows:

$$\begin{array}{cc} & Monomeric\;anthocyanin\;pigment\;(mg/100\;\;mL)\\ & =\left(A\times MW\times DF\times 1000\right)/\left(\varepsilon \times 1\right) \end{array}$$

where: MW (molecular weight) = 449.2; DF (dilution factor) = 5; ɛ (absorptivity) = 26,900.

#### 2.4.2 Total Phenolic Content—TPC

TPC was determined using the Folin‐Ciocalteu colorimetric assay, according to Mani, Johson, and Naiker [21], optimized for 96‐well microplates. Absorbance was measured at 760 nm using a Thermo Scientific Multiskan GO microplate reader (Waltham, MA, USA). Quantification was based on calibration curve constructed from gallic acid standards (CAS 149‐91‐7, 99 %, Sigma‐Aldrich) ranging from 10 to 120 mg/L. Results were expressed in mg of gallic acid equivalent (GAE) per mL.

#### 2.4.3 Antioxidant Activity—FRAP (Ferric Reducing Antioxidant Power) and ORAC (Oxygen Radical Absorbance Capacity)

The assays followed Arruda et al. (2023) [22]. Trolox (CAS 53188‐07‐1, 99 %, Sigma‐Aldrich) was the calibration standard (25–400 µmol/L). FRAP [22] results were expressed as µmol Trolox equivalents per mL, while ORAC was expressed as µmol Trolox equivalents per mL, based on fluorescence decay area.

### 2.5 pH‐Controlled Batch Culture Systems

#### 2.5.1 Preparation of Stool Samples

Faecal samples were donated by three healthy individuals who followed inclusion criteria. Samples were collected and kept in a freezer. At the analysis moment, it was thawed and diluted 1:10 (w/v) with 0.1 mol/L of phosphate buffer solution (PBS) at pH 7.4 and then homogenized in a Stomacher for 2 min. This solution was then used in the experiment [23].

#### 2.5.2 Batch Culture Fermentation System

Three‐stage batch culture gut model system was applied to replicate the physicochemical conditions in the colon, where the pH was maintained at 6.8 [11, [24]. Six vessels were used to test the following: 1) FK (1% v/v); 2) natural kombucha (1% v/v); 3) unfermented tea (1% v/v); 4) commercial kombucha (1% v/v); 5) positive control (inulin, 1% w/v); and 6) negative control, without any substrate. Doses used correspond to physiological amount of the beverage and do not represent toxicity. The experiment was conducted three times, each time using a stool sample from a different donor. 2.5‐mL samples were collected at four timepoints (0, 4, 8, and 24 h). Supernatant obtained after centrifugation was maintained for the measurement of SCFA and BCFA, and the pellet was resuspended in glycerol/PBS (1:1 v/v) for microbial DNA extraction and profiling. Samples were kept at ‐80°C until further analysis. The sample size was n = 3, representing independent biological replicates.

#### 2.5.3 Analysis of SCFA and BCFA

Acetic acid (C2), propionic acid (C3) and butyric acid (C4), and BCFA were analyzed using an ultra‐performance liquid chromatography‐tandem mass spectrometry (UPLC‐MS/MS) system, consisting of an Aquity UPLC H‐Class coupled to a Xevo TQ‐S micro‐ESI mass spectrometer (Waters, Wilmslow, UK). The standard solutions were purchased from Merck (Darmstadt, Germany).

The derivatization procedure was conducted using only the supernatant, following the method described by Valdivia‐Garcia et al. [25] in a 96‐well plate. Chromatographic separation was carried out on a HSS T3 analytical column (1.8 µm, 2.1 × 100 mm) in combination with an HSS T3 VanGuard pre‐column (Waters), maintained at 45°C. The mobile phase comprised solvent A (water with 0.1% formic acid) and solvent B (acetonitrile with 0.1% formic acid), operated at a flow rate of 0.6 mL/min.

The gradient program was initiated with 99% A for 1 min, followed by a linear transition to 5% A over 5 min, maintained at 5% A until 6.5 min, then re‐equilibrated to 99% A at 6.55 min and held constant until 8 min. MS/MS analysis was performed in positive ionization mode, applying an ionization voltage of 3.5 kV. The gas flow rate was set to 650 L/h at a temperature of 450°C. Transition parameters were referenced from Valdivia‐Garcia et al. [25].

#### 2.5.4 Gut Microbiota Profiling Through 16S rRNA Amplicon Sequencing

Microbial DNA was extracted from the pellet obtained after centrifugation (10 000 rpm for 15 min) of fermentation samples using the QIAamp DNA Stool Mini Kit (QIAGEN, Hilden, Germany), following the manufacturer's guidelines. DNA was quantified using a NanoDrop ND‐1000 spectrophotometer (Nano Drop Technologies, Wilmington, DE, USA).

For 16S rRNA amplicon sequencing, PCR amplification of the hypervariable V3‐V4 regions was performed using the S‐D‐Bact‐0341‐b‐S‐17/S‐D‐Bact‐0785‐a‐A‐21 primers [26] incorporating Illumina overhang adapter sequences as per the manufacturer's instructions. Sequencing was conducted on an Illumina MiSeq platform using a 2×250 bp paired end protocol according to manufacturer's specifications (Illumina, San Diego, CA, USA). Sequencing yielded a mean of 41 347 ± 15 610 reads per sample (range: 4888–75 855).

Raw sequences were processed using a pipeline combining PANDASeq [27] and QIIME 2 [28]. Following quality filtering and denoising in QIIME 2, samples were rarefied to 2342 reads per sample, when rarefaction curves reached a plateau. The filtered reads were grouped into amplicon sequence variants (ASVs) using DADA2 [29, 30]. Taxonomic assignment was performed using the VSEARCH algorithm [29] against the SILVA database [31]. Alpha diversity was assessed using several metrics, namely the Shannon index, the number of observed features and Faith's phylogenetic diversity (PD). Beta diversity was computed based on weighted and unweighted UniFrac distances, as well as Bray‐Curtis dissimilarity, and visualized on a Principal Coordinates Analysis (PCoA) plot.

### 2.6 Statistical Analysis

All data, except for the microbiota data, were analyzed using Analysis of Variance (ANOVA), Tukey's test or Student's t test, for comparison of means at a 5% significance level, using the software R (R Core Team, 2024).

For microbiota, statistical analyses were conducted using RStudio 2025.05.1+513 on R software version 4.5.1 (https://www.r‐project.org) implemented with the R packages stats and vegan (https://cran.r‐project.org/web/packages/vegan). The significance of data separation in the PCoA plot was tested by PERMANOVA using the adonis function in the vegan package. Differences in alpha diversity and relative taxon abundance were assessed using Kruskal‐Wallis tests followed by post‐hoc Wilcoxon tests. Paired Wilcoxon signed‐rank tests were applied for within‐group comparisons across timepoints, using donor as the pairing unit. Unpaired Wilcoxon rank‐sum tests were used for between‐group comparisons at each timepoint. The three faecal donors were treated as independent biological replicates. Timepoints were analyzed independently rather than modelled as repeated longitudinal data. A *p*‐value ≤ 0.05 was considered statistically significant, while a p‐value between 0.05 and 0.1 was considered to indicate a trend [32, 33]. The latter should be interpreted as exploratory and hypothesis‐generating rather than as conclusive.

## 3 Results and Discussion

### 3.1 Microbiological Analysis—Survival of Yeasts, AAB, and LAB in FK Under in Vitro Digestion

Viable counts of flavored kombucha (FK) following simulated in vitro digestion are presented in Table 1. The microbiological assessment was deliberately restricted to the enumeration of major functional groups, LAB, AAB, and yeasts, to evaluate microbial resilience under gastrointestinal conditions, rather than to resolve taxonomic composition.

**TABLE 1:** Viability of yeasts, AAB, and LAB in FK during in vitro digestion. Results are presented as the mean ± standard deviation of CFU/mL.

Sample	Viability (log10 CFU/ml)		
	Yeasts	AAB	LAB
*Flavored kombucha*	7.65 ± 0.02 ^A^	7.49 ± 0.03 ^A^	7.47 ± 0.13 ^A^
*Salivary phase*	7.35 ± 0.12 ^A^	7.51 ± 0.32 ^A^	7.16 ± 0.06 ^B^
*Gastric phase*	7.18 ± 0.40 ^A^	7.05 ± 0.39 ^A^	6.97 ± 0.46 ^B^
*Small intestinal phase*	4.58 ± 0.72 ^B^	5.09 ± 0.71 ^B^	4.22 ± 0.23 ^C^

^A‐B^ Means with different letters within a column differ significantly (*p* < 0.05, Tukey's test).

Overall, these microbial groups exhibited tolerance to salivary and gastric phases, with only minor reductions, particularly for LAB. A more pronounced decline in viability was observed during the intestinal phase, likely associated with bile and enzymatic stress. Despite this, viable populations remained above 4.2 log_10_ CFU/mL, indicating partial survival of the microbial consortium under the applied conditions.

Importantly, these findings refer to group‐level survival and do not provide taxonomic or strain‐specific resolution, as no post‐digestion identification was performed. This approach is consistent with in vitro studies aimed at assessing the persistence of functional microbial consortia, without inferring colonization capacity or host‐related effects. In line with previous reports [34, 35], kombucha‐associated microorganisms can remain viable after simulated digestion; however, such observations should be interpreted strictly as indicators of survival potential.

Collectively, the data suggest that kombucha‐associated microbial consortia display a degree of resilience to gastrointestinal stress in vitro. Further studies integrating taxonomic profiling and in vivo validation are required to determine their functional relevance within the host.

### 3.2 Bioaccessibility of Bioactive Compounds

Antioxidant capacity, anthocyanins, and TPC of FK were evaluated in the beverage and during simulated gastrointestinal digestion (Table 2). During the small intestinal phase of digestion, the values decreased corresponding to a bioaccessibility of 31.38% and 2.79%, respectively. Some studies have reported a reduction in antioxidant capacity following gastrointestinal digestion of foods [12, 36], whereas others have suggested that intestinal digestion may enhance it [37]. The decrease is explained by the acidic or alkaline conditions of the gastrointestinal environment, causing the removal of hydroxyl groups from phenolic compounds [38], whereas the increase may result from the generation of novel compounds or the modification of precursor molecules, both of which exhibit elevated antioxidant capacity [39]. Overall, our data are in line with Degirmencioglu et al. [37], who found that bioaccessibility values of antioxidant capacity decreased comparing predigestion to postdigestion samples.

**TABLE 2:** Functional characteristics of FK before and during in vitro digestion. Anthocyanins, TPC, and antioxidant capacity (ORAC and FRAP). Results are presented as mean ± standard deviation and bioaccessibility (%).

Treatment	Anthocyanins (mg.100mL^−1^)	TPC (mg GAE.100mL^−1^)	ORAC (µmol TE.100mL^−1^)	FRAP µmol TE.100mL^−1^)
Flavored Kombucha	2.23^A^ ± 0.34	80.59^A^ ± 3.46	866.05^A^ ± 53.77	606.83^A^ ± 55.83
Salivary phase	0.87^B^ ± 0.04	28.03^B^ ± 0.83	418.74^B^ ± 42.89	98.96^B^ ± 4.49
Gastric phase	1.45^C^ ± 0.21	22.01^C^ ± 0.44	387.92^B^ ± 39.96	98.22^B^ ± 8.60
Small intestinal phase	0.12^D^ ± 0.04	16.89^D^ ± 0.21	270.94^C^ ± 10.50	16.93^C^ ± 1.38
Bioaccessibility (%)	39.27	20.95	31.38	2.79

^A‐D^ Means with different letters within a column differ significantly (*p* < 0.05, Tukey's test).

For anthocyanin, a bioaccessibility of 39.27% was reached at the end of intestinal phase and for TPC a bioaccessibility of 20.95%. As mentioned above, bioaccessible phenolic compounds are susceptible to external conditions, which may influence their stability and promote interactions with other food constituents, thereby explaining between‐study differences in bioaccessibility [40]. Catechins derived from tea exhibit limited stability when exposed to neutral or alkaline pH environments, where their integrity is compromised by oxidative conditions and the presence of metal ions, while flavonols are generally considered to be more resistant, although they may still undergo partial breakdown during gastrointestinal processes [41].

In particular, within the food matrix, polyphenolic compounds may either interact with other constituents or undergo structural degradation resulting from hydrolysis and enzymatic reactions [13, 38]. PH fluctuations and the action of enzymes in the digestive process directly influence the bioaccessibility of phenolics. As a result, reduced amounts of phenolic compounds are available in the colon [37, 41]. The variation in antioxidant capacity observed between studies could be attributed to the distinct methods used [12].

### 3.3 In Vitro Colonic Fermentation

#### 3.3.1 Analysis of SCFAs and BCFAs

In general, SCFA levels increased during 24‐h fermentation (Table 3). FK exhibited a pronounced increase in acetate after 24 h, accompanied by concomitant increases in butyrate, and BCFAs. However, there was considerable variability at later stages of fermentation, which could be explained by the different faecal donors. Greater standard deviation is observed in some results after 24‐h fermentation, due to an exceeded production on fatty acids in the first experiment, probably originating from the microbiota of faecal donor. Statistical differences in the acetate production separated the positive control, flavored and natural kombucha, to the tea, commercial kombucha and the negative control. It shows that even with the dependence of the donor microbiota, these fermented beverages represent potential substrate for the production of important SCFAs. Natural kombucha exhibited a similar albeit less marked pattern, with acetate and BCFAs increasing after 24 h. By contrast, tea and commercial kombucha showed minimal changes in all metabolites. These findings are consistent with those of Linhares et al. [13], who observed increased levels of butyrate, propionate and acetate after 48 h of in vitro colonic fermentation of different types of kombucha.

**TABLE 3:** Levels of short‐chain fatty acids (acetate, propionate, and butyrate) and branched‐chain fatty acids (BCFAs) in in vitro batch culture systems. Concentrations (mM.L^−1^) were determined at 0, 4, 8, and 24 h of fermentation of different substrates (FK, natural kombucha, tea, commercial kombucha, positive (inulin), and negative control) in stirred pH‐controlled batch culture systems with stool samples from healthy donors. Data are presented as mean ± standard deviation (n = 3, independent biological replicates).

Treatment	Time (h)	Acetate	Butyrate	Propionate	BCFAs
FK	0	1.587 ± 0.11	0.166 ± 0.06	0.069 ± 0.06	0.205 ± 0.10
	4	2.017 ± 1.43	0.226 ± 0.13	0.071 ± 0.02	0.177 ± 0.05
	8	3.904 ± 2.97	0.570 ± 0.51	0.079 ± 0.05	0.259 ± 0.15
	24	10.074^A^ ± 11.30	1.644 ^A^ ± 2.16	0.172 ^A^ ± 0.19	4.118 ^A^ ± 4.18
Natural Kombucha	0	1.368 ± 0.21	0.165 ± 0.05	0.067 ± 0.05	0.204 ± 0.10
	4	1.583 ± 0.81	0.212 ± 0.09	0.060 ± 0.05	0.182 ± 0.09
	8	3.054 ± 1.45	0.437 ± 0.27	0.058 ± 0.05	0.379 ± 0.31
	24	3.01^A^ ± 2.74	0.284 ^A^ ± 0.23	0.054 ^A^ ± 0.05	1.180 ^A^ ± 1.92
Tea	0	1.127 ± 0.22	0.153 ± 0.04	0.056 ± 0.05	0.186 ± 0.06
	4	0.770 ± 0.08	0.131 ± 0.03	0.041 ± 0.04	0.122 ± 0.05
	8	1.051 ± 0.15	0.146 ± 0.05	0.054 ± 0.06	0.160 ± 0.11
	24	2.123^*^, ^B^ ± 0.93	0.178 ^A^ ± 0.04	0.053 ^A^ ± 0.05	0.150 ^A^ ± 0.10
Commercial Kombucha	0	1.123 ± 0.28	0.145 ± 0.04	0.051 ± 0.05	0.170 ± 0.07
	8	0.777 ± 0.20	0.128 ± 0.04	0.038 ± 0.04	0.119 ± 0.08
	4	1.200 ± 0.64	0.173 ± 0.08	0.041 ± 0.04	0.120 ± 0.08
	24	1.761^B^ ± 1.35	0.194 ^A^ ± 0.09	0.050 ^A^ ± 0.05	0.144 ^A^ ± 0.10
Positive control	0	1.213 ± 0.16	0.162 ± 0.05	0.062 ± 0.05	0.220 ± 0.08
	4	1.322 ± 0.59	0.152 ± 0.02	0.050 ± 0.03	0.168 ± 0.04
	8	3.384^*^ ± 2.57	0.209 ± 0.11	0.046 ± 0.03	0.152 ± 0.05
	24	12.785^*^, ^A^ ± 10.73	0.410 ^A^ ± 0.41	0.347 ^A^ ± 0.50	0.185 ^A^ ± 0.05
Negative control	0	1.065 ± 0.25	0.169 ± 0.07	0.079 ± 0.08	0.231 ± 0.16
	4	1.037 ± 0.62	0.161 ± 0.07	0.072 ± 0.08	0.196 ± 0.18
	8	1.694 ± 1.40	0.265 ± 0.24	0.076 ± 0.10	0.191 ± 0.18
	24	1.952^B^ ± 1.28	0.276 ^A^ ± 0.23	0.077 ^A^ ± 0.09	0.192 ^A^ ± 0.18

^A‐B^ Significant differences of each SCFA at 24 h of fermentation using two‐way ANOVA with Tukey's post‐hoc comparison (*P*< 0.05).

^*^ Significant differences compared to baseline (0 h) within the same substrate (using t‐test, *P*< 0.05).

The increased BCFA levels observed in the FK may reflect changes in the substrate composition and active microbial metabolism during fermentation rather than an exclusively detrimental effect. Because BCFA production occurred alongside SCFA formation, these results suggest the coexistence of proteolytic and saccharolytic fermentation pathways. Recent studies indicate that kombucha fermentation involves complex microbial interactions and dynamic nitrogen metabolism within the SCOBY consortium, supporting the interpretation. Nevertheless, excessive proteolytic fermentation can be associated with less favorable metabolic outcomes, further investigation of nitrogen metabolism and temporal metabolite dynamics is warranted.

#### 3.3.2 Impact on Gut Microbiota

The sequencing reads were deposited in the National Center for Biotechnology Information Sequence Read Archive (Bioproject ID: PRJNA1476124). Overall, alpha diversity tended to decreased over time (p ≤ 0.1, Wilcoxon test) (Figure 1), consistent with closed fermentation dynamics in which substrate depletion enriches fast‐growing taxa [42]. FK showed reduced diversity at all timepoints versus baseline, indicating rapid substrate use and accelerated fermentation‐driven ecological collapse. Conversely, commercial kombucha remained stable, with no significant temporal changes. Natural kombucha showed a milder decline, with reduced diversity only after 24 h (observed features and Faith's PD), while tea showed no differences. These results suggest FK exerts the greatest and fastest modulatory effect on gut microbiota, likely due to added fermentable substrates and polyphenols from forest fruits [43, 44, 45, 46]. While this indicates high microbial metabolic activity, it may also reflect a less stable microbial community structure. Decreased diversity is generally considered an unfavorable feature of gut ecosystems, as it is associated with poor resilience and impaired host health [47].

**FIGURE 1:**
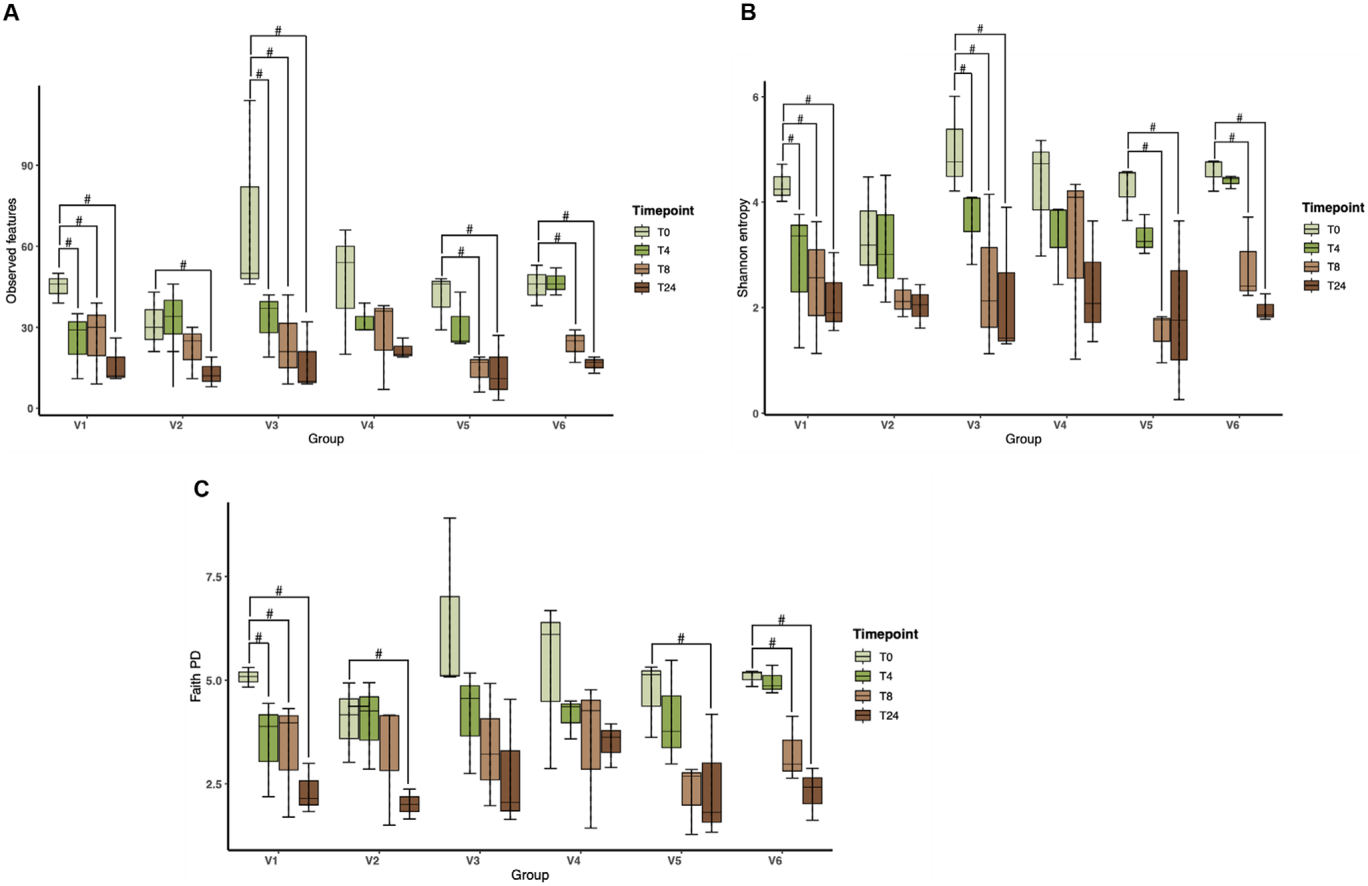
Alpha diversity of the gut microbiota in in vitro batch culture systems. Box and whisker plots showing the median (center line), the 25^th^ to 75^th^ percentile (box) and the range (whiskers) of alpha diversity, measured according to the number of observed features (A), the Shannon index (B) and Faith's Phylogenetic Diversity (C), at 0, 4, 8, and 24 h of fermentation of different substrates (FK—V1, natural kombucha—V2, tea—V3, commercial kombucha—V4, positive (inulin)—V5 and negative control—V6) in stirred pH‐controlled batch culture systems with stool samples from healthy donors. # 0.05 ≤ *p* ≤ 0.1, Wilcoxon test (n = 3, independent biological replicates).

Beta diversity analysis (weighted UniFrac and Bray‐Curtis) showed significant sample segregation (*p* ≤ 0.02, PERMANOVA) (Figure 2), mainly driven by time, with 24 h samples clustering separately. Condition‐specific differences were also observed: unflavored kombucha differed from tea, commercial kombucha, and the negative control (weighted UniFrac), while flavored and commercial kombucha differed from the negative control (Bray‐Curtis) (*p* ≤ 0.05). No separation was found with unweighted UniFrac or Jaccard (p ≥ 0.2) (data not shown). Overall, time was the main driver of diversification, but kombucha formulations differentially affected microbiota structure, particularly dominant taxa.

**FIGURE 2:**
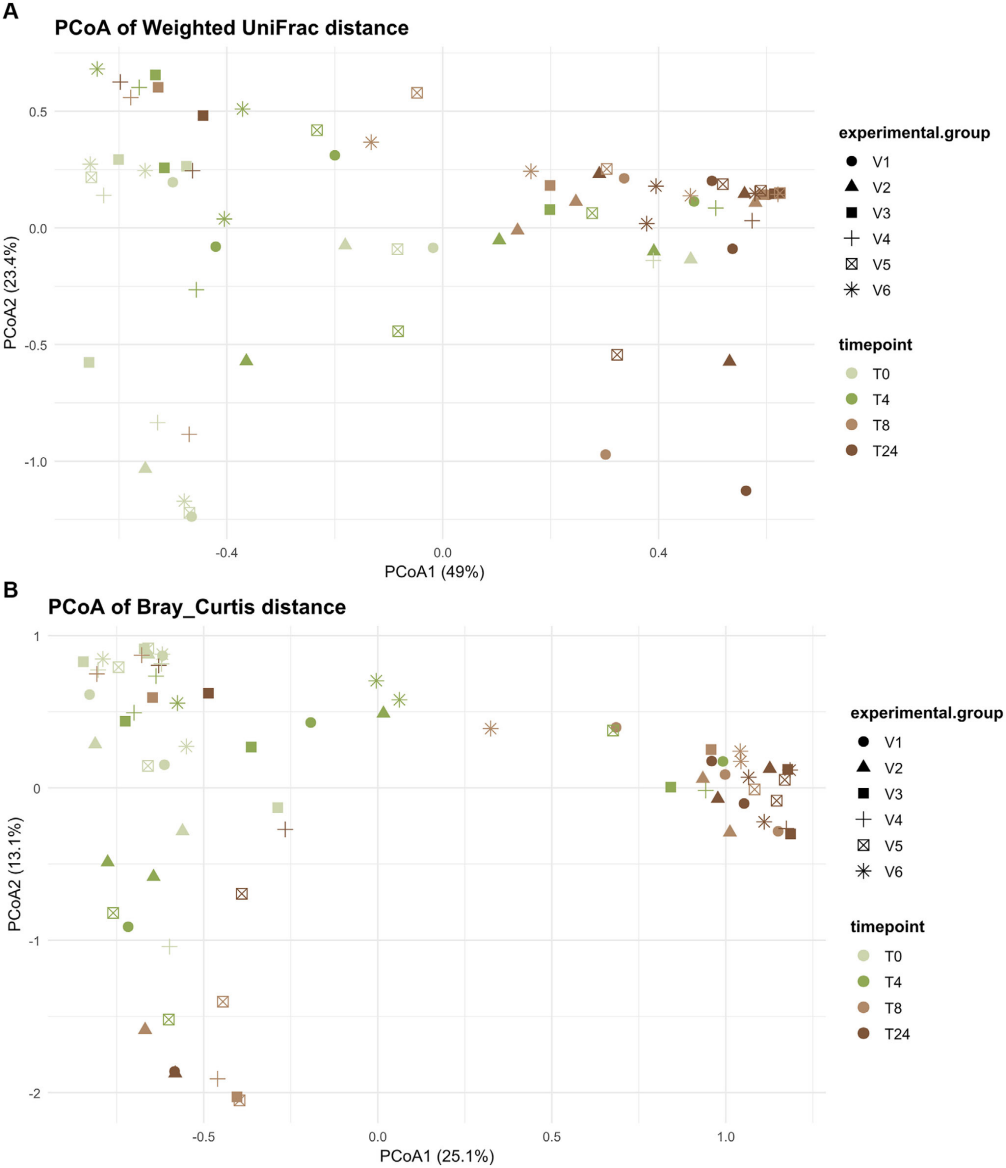
Beta diversity of gut microbiota in in vitro batch culture systems. Principal Coordinates Analysis (PCoA) based on weighted UniFrac (A) and Bray‐Curtis (B) distances between gut microbiota profiles at 0, 4, 8, and 24 h of fermentation of different substrates (FK—V1, natural kombucha—V2, tea—V3, commercial kombucha—V4, positive (inulin)—V5 and negative control—V6) in stirred pH‐controlled batch culture systems with stool samples from healthy donors. A significant separation was found (*p* ≤ 0.02, PERMANOVA) (n = 3, independent biological replicates).

Taxonomically, at baseline, all samples showed similar composition, dominated by the phyla Bacillota (52.3%), Pseudomonadota (25.4%), and Bacteroidota (15.0%), and the families *Ruminococcaceae* (16.5%), *Lachnospiraceae* (14.1%), and *Burkholderiaceae* (12.7%) (Figure 3). During fermentation, family‐and genus‐level shifts occurred (*p* ≤ 0.1, Wilcoxon test) (Figures 4 and 5, respectively). *Enterobacteriaceae*, particularly *Escherichia‐Shigella*, increased over time with all substrates except commercial kombucha, which maintained relative stability up to 24 h. Although these taxa are known for their metabolic versatility and rapid growth in in vitro gut fermentation models [48], their expansion is frequently interpreted as a marker of dysbiosis or substrate imbalance, particularly in conditions with excess of simple carbohydrates—as is the case of kombucha—or disrupted microbial competition. This bloom may also explain both the reduction in alpha diversity and the clustering patterns observed in beta diversity analyses, as previously discussed [45].

**FIGURE 3:**
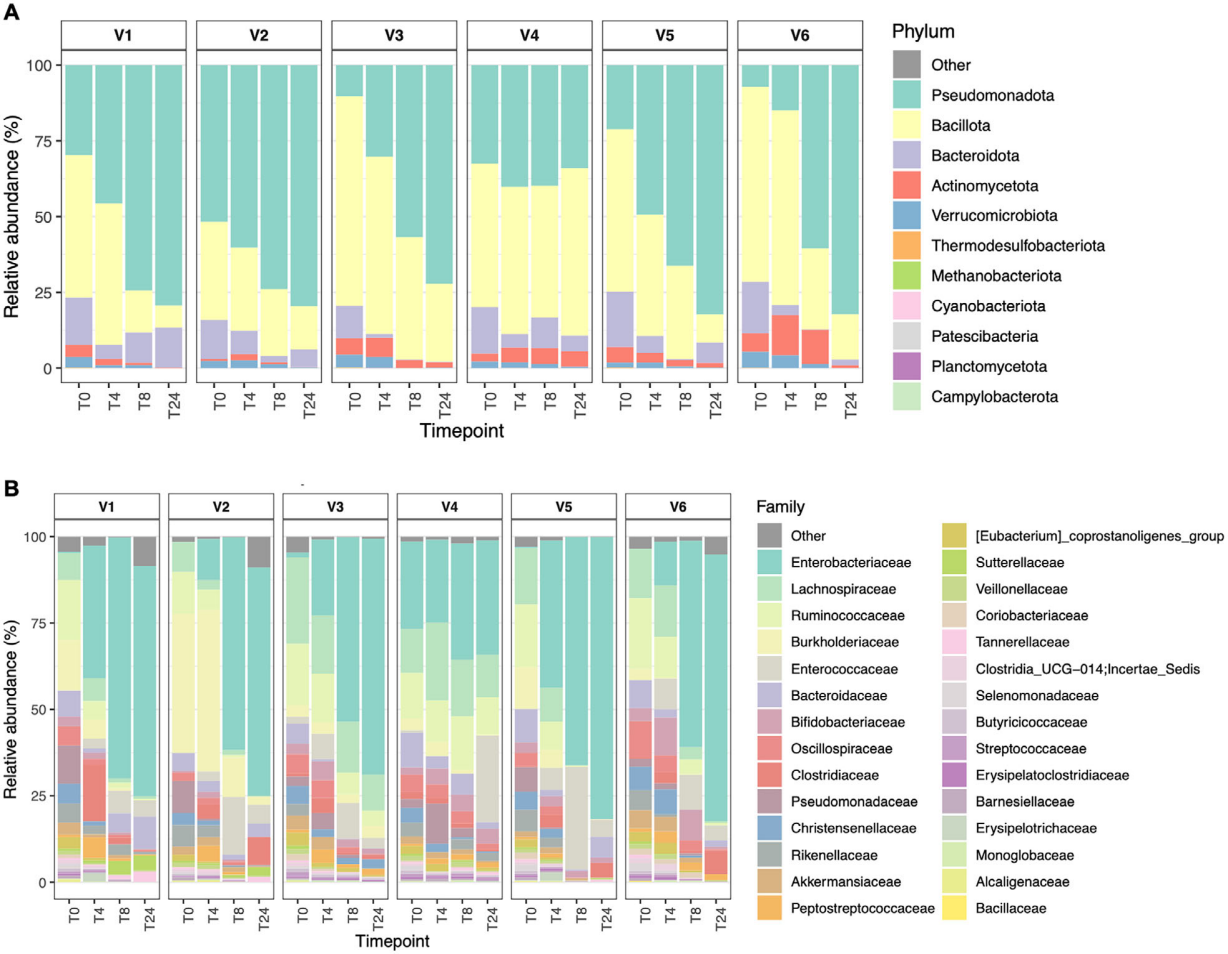
Gut microbiota composition in in vitro batch culture systems. Bar plots showing relative abundance of different taxa at the phylum (A) and family (B) level at 0, 4, 8, and 24 h of fermentation of different substrates (FK—V1, natural kombucha—V2, tea—V3, commercial kombucha—V4, positive (inulin)—V5 and negative control—V6) in stirred pH‐controlled batch culture systems with stool samples from healthy donors. Only taxa with relative abundance >0.1% in at least 1 sample are shown (n = 3, independent biological replicates).

**FIGURE 4:**
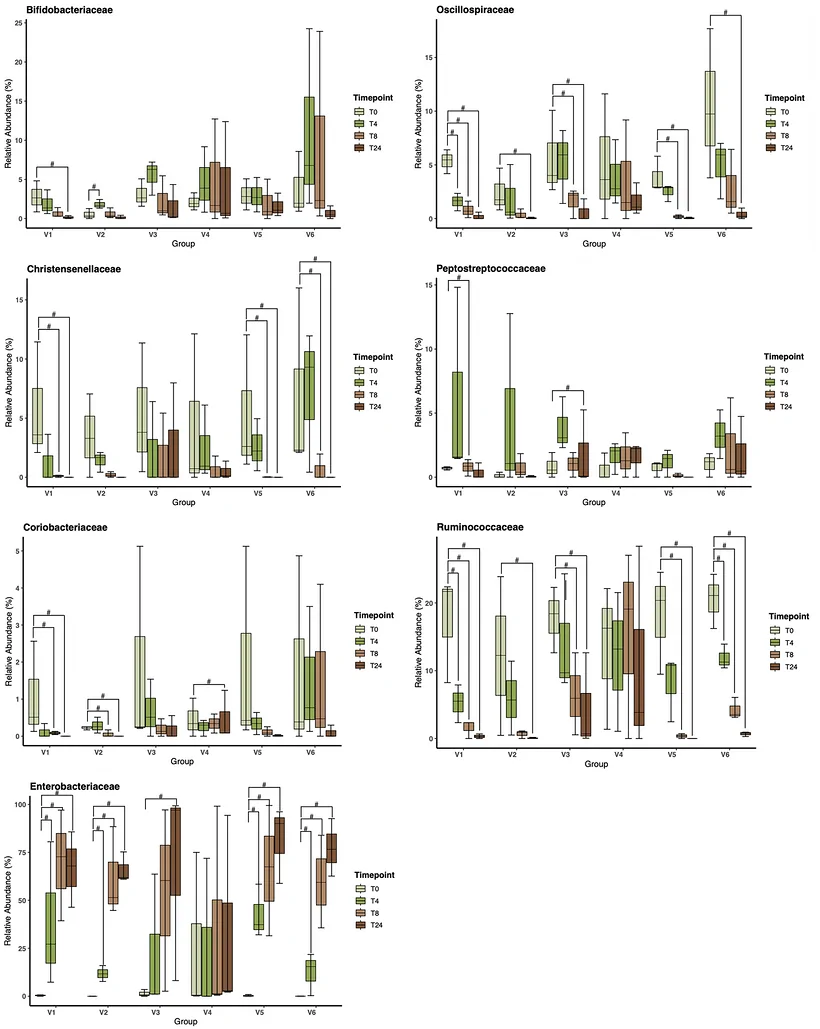
Family‐level differences in the gut microbiota in in vitro batch culture systems. Box and whisker plots showing the median (center line), the 25^th^ to 75^th^ percentile (box) and the range (whiskers) of the relative abundance of families differentially represented in the gut microbiota at 0, 4, 8, and 24 h of fermentation of different substrates (FK—V1, natural kombucha—V2, tea—V3, commercial kombucha—V4, positive (inulin)—V5 and negative control—V6) in stirred pH‐controlled batch culture systems with stool samples from healthy donors. # 0.05 ≤ *p* ≤ 0.1, Wilcoxon test (n = 3, independent biological replicates).

**FIGURE 5:**
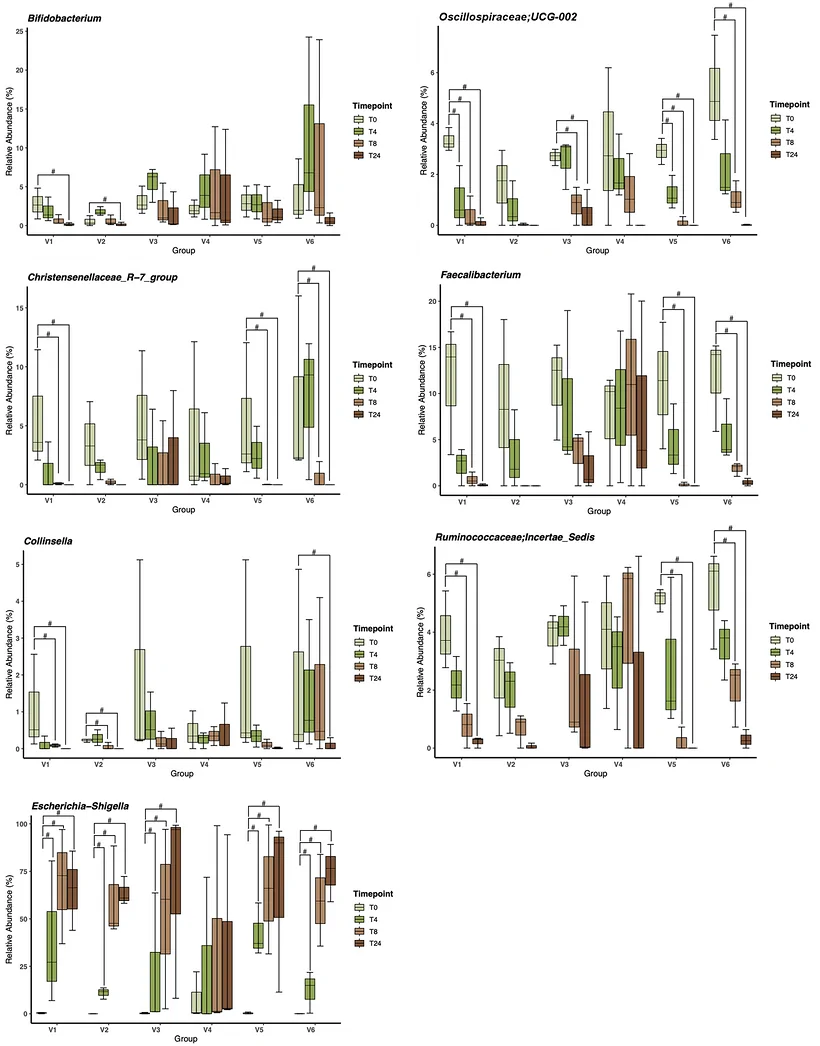
Genus‐level differences in the gut microbiota in in vitro batch culture systems. Box and whisker plots showing the median (center line), the 25^th^ to 75^th^ percentile (box) and the range (whiskers) of the relative abundance of genera differentially represented in the gut microbiota at 0, 4, 8, and 24 h of fermentation of different substrates (FK—V1, natural kombucha—V2, tea—V3, commercial kombucha—V4, positive (inulin)—V5 and negative control—V6) in stirred pH‐controlled batch culture systems with stool samples from healthy donors. # 0.05 ≤ *p* ≤ 0.1, Wilcoxon test (n = 3, independent biological replicates).

FK induced the most changes, with decreases in Bifidobacteriaceae (Bifidobacterium), Christensenellaceae (R7 group), Coriobacteriaceae (Collinsella), Oscillospiraceae (UCG‐002), and Ruminococcaceae (Faecalibacterium, Incertae sedis), and an increase in Peptostreptococcaceae. Natural kombucha also reduced Bifidobacterium and Coriobacteriaceae (Collinsella), the latter likewise decreasing with commercial kombucha, indicating formulation‐specific effects.

The FK‐related reduction in several taxa generally considered beneficial, particularly *Bifidobacterium* and *Faecalibacterium*, which are associated with host health due to their roles in SCFA production, gut barrier maintenance and anti‐inflammatory activity [49, 50], may be unfavorable. This decrease suggests that, despite stimulating overall SCFA production, FK may not selectively promote canonical health‐promoting taxa. Conversely, the reduction of *Coriobacteriaceae* may be favorable, as *Collinsella* is an obesogenic taxon linked to increased energy harvest, altered bile acids and lipid metabolism, gut permeability, and inflammation [42]. This may help to explain the potential anti‐obesity effects of kombucha [51]. However, bearing in mind the simultaneous expansion of *Enterobacteriaceae*, as discussed above, these effects should be interpreted cautiously.

Decreases in *Christensenellaceae*, *Oscillospiraceae*, and *Ruminococcaceae* with FK were also seen in controls, suggesting model‐related variation. *Lachnospiraceae* decreased only in controls, suggesting kombucha and tea help preserve this key SCFA‐producing family. In contrast, *Bacteroidaceae* (*Bacteroides*) declined with tea, commercial kombucha, and the negative control, possibly explaining the acetate increase seen with flavored and natural kombucha [52]. Commercial kombucha and tea also reduced *Rikenellaceae* (*Alistipes*), an acetate producer previously shown to decrease after green tea kombucha intake in overweight individuals [51, 53]. Other changes were inconsistent with current literature, likely due to limited and heterogeneous studies [54]. Although SCOBY taxa (e.g., *Acetobacter*, *Komagateibacter*, and *Liquorilactobacillus*) [55] were not detected, possibly due to being below detection limits, this suggests kombucha acts indirectly, modulating microbiota without persistent engraftment [56].

Taken together, these findings indicate that kombucha, particularly the flavored formulation, acts as a fermentable substrate that has the potential to modulate the composition of the gut microbiota. However, given the simultaneous enrichment of potentially undesirable taxa and reduction of established beneficial genera, as well as the inherent constraints of in vitro modelling, these findings should be interpreted cautiously and further validated before any conclusions regarding gut health can be drawn.

Even though flavored kombucha induced distinct changes in gut microbiota composition and microbial metabolite production, these findings cannot be directly attributed to specific physicochemical characteristics of the beverage, as a physicochemical characterization was not available. Therefore, any mechanistic interpretation of the relationship between beverage composition and the observed microbial responses should be considered exploratory and suggestive rather than conclusive.

## 4 Concluding Remarks

Using a static in vitro gastrointestinal digestion and colonic fermentation model, this exploratory study provides evidence that kombucha may influence gut microbial activity, as indicated by changes in short‐chain fatty acid (SCFA) production. Flavored kombucha prepared with native fruits showed indications of modulating microbial layout and contributing to functional properties under the experimental conditions applied. However, it should be noted that some compositional changes were unfavorable, such as the increase in enterobacteria over time and the corresponding decrease in certain beneficial taxa, including *Bifidobacterium*. Though, these observations are restricted to the in vitro model and should not be directly extrapolated to in vivo physiological effects.

The focus on flavored kombucha for bioactive compound analyses was aligned with the objective of assessing the impact of flavoring and enrichment strategies on the beverage's functional profile. Natural and commercial kombucha samples were included as references for fermentation behavior and microbiological comparisons. Future studies incorporating comprehensive bioactive profiling across all sample types would allow a more robust functional comparison.

The main limitations of this study are as follows: i) different viable microorganisms present in FK and during in vitro digestion, were not taxonomically identified, which prevented more accurate characterization of the produce and limited interpretation of the gut microbiota data; ii) viable plate counting and assays of the functional characteristics (anthocyanins, TPC, and antioxidant capacity) of FK did not use unflavored and/or commercial counterparts for comparison; and iii) the small number of donors used and the high variability in their gut microbiota composition (an obvious limitation of an in vitro study) limited the statistical power; and iv) the lack of a physicochemical characterization of the kombucha formulations prior to the in vitro colonic fermentation experiments. However, available studies suggest kombucha has modest capacity to modulate the gut microbiota. Further research is needed to clarify its mechanisms and long‐term effects.

## Conflicts of Interest

The authors have no conflicts of interest to declare.

## Data Availability

The data that support the findings of this study are available from the corresponding author upon reasonable request.
